# Spawning energetics and otolith microchemistry provide insights into the stock structure of bonga shad *Ethmalosa fimbriata*

**DOI:** 10.1111/jfb.13881

**Published:** 2019-01-15

**Authors:** Julian Döring, Carola Wagner, Maik Tiedemann, Patrice Brehmer, Werner Ekau

**Affiliations:** ^1^ Leibniz Centre for Tropical Marine Research (ZMT), Department of Ecology Bremen Germany; ^2^ Institute of Marine Research, Pelagic Fish Bergen Norway; ^3^ Institut de Recherche pour le Développement (IRD), UMR Lemar (CNRS, UBO, IRD, Ifremer) Dakar Senegal; ^4^ Centre de Recherche Océanographique de Dakar‐Thiaroye (CRODT) Institut Sénégalais de Recherche Agricole (ISRA) Pôle de Recherche de Hann Senegal

**Keywords:** Clupeidae, inverse estuary, oocyte, reproductive investment, Senegal, stock discrimination, spawning components

## Abstract

The gross energy content of spawning batches and the microchemistry of sagittal otoliths in individual female bonga shad *Ethmalosa fimbriata* were compared between contrasting sampling sites at the Senegalese southern coast and inside the hypersaline Sine Saloum Estuary. Results show that females spawning in the estuary's middle reaches invested almost three times more energy into reproduction (115 ± 65 J g^−1^ body mass) than their neritic counterparts (39 ± 34 J g^−1^ body mass). Also, female otolith levels of Ba:Ca, Sr:Ca and Zn:Ca either differed significantly between study sites or could be linked to heterogeneous environmental variables. A quadratic discriminant function analysis provided evidence of segregated spawning populations of *E. fimbriata* in southern Senegalese waters.

## 1 INTRODUCTION

The bonga shad *Ethmalosa fimbriata* (Bowdich 1825) is an exploited clupeid that occurs along the West African coast from Mauritania to Angola (Charles‐Dominique & Albaret, 2003). In Senegal, the species’ fisheries landings totalled almost 17,000 t in 2014, making it one of the region's economically most important clupeids (Ndaw *et al*., 2015). Previous studies suggest that *E. fimbriata* is locally adapted with regard to its morphology, growth and reproductive traits (Charles‐Dominique & Albaret, 2003). Differences observed in life‐history patterns indicate that West African populations are largely separated and only a few individuals are suspected to migrate between habitats (Charles‐Dominique, 1982; Panfili *et al*., 2006). A study by Durand *et al*. (2013) identified genetic differences between specimens sampled in West African oceanic waters (Guinea, Banc d’Arguin in Mauritania) and individuals sampled inside estuaries (Sine Saloum Estuary Gambia and Casamance Rivers), attributing heterogeneous genetic patterns to alternating life cycles and to the species’ occurrence in estuaries with distinct hydrological features. It is worth noting, however, that no individuals from southern Senegalese shelf waters were sampled.

At the Senegalese southern coast *E. fimbriata* inhabits cold upwelling waters as well as extremely warm waters in the Sine Saloum, a mangrove estuary severely affected by long‐term climatic changes (Charles‐Dominique, 1982; Panfili *et al*., 2004). Until the 1970s, this estuary used to be species‐rich and supported a high‐yielding fishery. Owing to a decline in precipitation and rising temperatures in the Sahel zone over past decades, this estuary transformed into an inverse system with a permanently reversed salinity gradient (Pagès & Citeau, 1990). The hypersaline conditions in the upper reaches of the estuary (salinity up to 130), as well as strong fishing pressure already led to an immense reduction in the overall annual catch rates of *c*. 50%–80% over the past 50 years (Pagès & Citeau, 1990; Simier *et al*., 2004; Villanueva, 2015). As effective fisheries management requires assessment of connectivity patterns among populations, small‐scale studies on the migration and dispersal of *E. fimbriata* in Senegalese waters are necessary to manage its stock more efficiently (Durand *et al*., 2013).

The term stock refers to a population that is reproductively self‐sustaining and comprises individual fish displaying identical life‐history traits as a response to environmental characteristics (Ihssen *et al*., 1981). Since certain traits may be expressed differently in the same genotype, as an adaptive response to environmental factors, stock discrimination solely on the basis of genetics is inadequate (Begg *et al*., 1999; MacLean & Evans, 1981; Swain & Foote, 1999). Small pelagic fish stocks are often composed of different spawning components and each component's contribution to the fished stock varies with its productivity (Begg *et al*., 1999). Generally, less productive components are more vulnerable to fishing pressure than more productive ones (Jennings *et al*., 1998) and their overexploitation may ultimately lead to a loss of genetic variability (Stephenson, 1999). To facilitate accurate stock assessment and to improve fisheries management plans, the relative contribution of a spawning component to the entire stock needs to be estimated (Hilborn, 1985).

The productivity of an entire stock is subject to variations in reproductive potential of individual females, which, in turn, is subject to the surplus energy available for spawning (Döring *et al*., 2018; Pecquerie *et al*., 2009). Hatching success or viability of early larval stages was found to be related to maternal investments, such as the mass of the oocyte and the amount of lipids and proteins deposited into the oocyte (Brooks *et al*., 1997; Kamler, 2005). An increase in egg‐lipid and protein content enhances hatching success in clupeids (Castro *et al*., 2009). Furthermore, elevated energy reserves (protein and lipid content) support larval condition by increasing the time window until first feeding has to be established (Guisande *et al*., 1998). Thus, knowledge on egg composition is essential, since it has direct consequences for early life‐stage survival. The number of viable offspring is also influenced by the number of produced oocytes; *i.e*., the batch fecundity in indeterminate spawners such as *E. fimbriata* (Rickman *et al*., 2000). An individual female's ability to invest energy into reproduction is therefore closely coupled with the stock's reproductive potential and with its productivity (Pecquerie *et al*., 2009).

Apart from determining a stock's productivity, the identification of spawning areas and migration patterns are crucial for developing sustainable fisheries management strategies (Colloca *et al*., 2009). Elemental analysis of fish otoliths is a widely used methodology for identifying spawning areas and discriminating among stock components (Avigliano *et al*., 2017; Kerr & Campana, 2014). The chemical composition of otoliths is a natural marker of habitat use due to the otolith's continuous growth throughout the fish's lifetime and the fact that the deposited otolith material is metabolically inert (Campana & Neilson, 1985). Physiological factors, as well as variations in water chemistry, can affect the incorporation of trace elements into the otolith (Campana, 1999). Therefore, the concentration of selected elements (the elemental fingerprint) in the otolith can be used as a natural tag to discriminate between groups of fish that have spent at least part of their lives in different environments (Kerr & Campana, 2014).

This study aims to assess the stock structure of *E. fimbriata* in southern Senegalese coastal waters based on spawning energetics–productivity of individual females and their otolith elemental fingerprint. For this purpose, the gross energy content of spawning batches and the microchemistry of sagittal otoliths were compared between one sampling site at the Senegalese southern coast, one inside the Sine Saloum Estuary and one at its mouth.

## 2 MATERIALS AND METHODS

### 2.1 Study area and environmental parameters

The Senegalese southern coast (SSC) or ‘Petite‐Côte’ (14° 36′–13° 36′N) is located in the southern part of the Canary Current large marine ecosystem, one of the most productive upwelling ecosystems in the world (Chavez & Messié, 2009). The SSC harbours a seasonal upwelling cell, which governs ambient temperature fluctuations in the region. Because of the shelf's topography, northerly trade winds induce a strong tongue‐shaped upwelling core in winter–spring (Capet *et al*., 2017; Ndoye *et al*., 2017). While in coastal regions with a narrow shelf the upwelling core is observed at the shelf break, the upwelling core at the SSC occurs on the shelf, where water depths are <100 m (Arístegui *et al*., 2009; Ndoye *et al*., 2014). In concert with sunlight, the upwelled cold and nutrient‐rich bottom waters facilitate the growth of phytoplankton which constitutes the basis of marine food webs (Auger *et al*., 2016).

**Figure 1:**
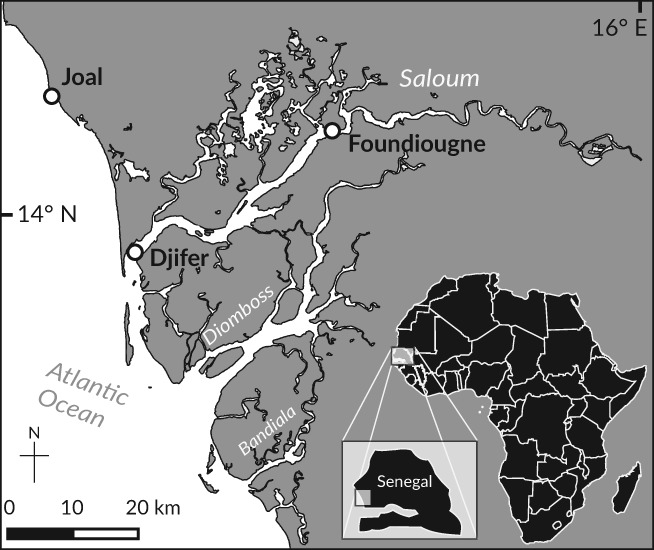
The Senegalese southern coast and the Sine Saloum Estuary, including sampling sites (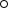): Joal, Senegalese southern coast; Djifer, Saloum River mouth; Foundiougne, Saloum River middle reaches

The Sine Saloum Estuary is located at the southern tip of the SSC and extends over an open water surface of *c*. 800 km^2^ (from 13° 55'–14° 10′ N to 16°03′–16° 50′ W; Figure 1). This coastal ecosystem is characterized by slight freshwater input from isolated groundwater discharge and small inflow of rivers, while rainfall is the main freshwater supply in the system. Its river system consists of three main branches: the Saloum, Diomboss and Bandiala. The water column of these main branches is well mixed and reaches a maximum depth of 15 m (Sloterdijk *et al*., 2017). The climate in the Sine Saloum region is characterized by a dry season (usually from November to June) and a short warm rainy season (usually from July to October) (Pagès & Citeau, 1990).

Satellite‐derived (moderate‐resolution imaging spectroradiometer; MODIS Aqua, level 2, 0.1°) sea surface temperatures were assessed at Joal (15 km radius) and Djifer once per sampling week. As no remote‐sensing data for inland waters are available, the Saloum River surface‐water temperatures were recorded *in situ* once per sampling week with a digital thermometer (ama‐digit ad 15th; precision 0.4%; accuracy 0.4%; Amarell; http://www.amarell.de). At Djifer and Foundiougne, salinity was determined once per sampling week according to the practical salinity scale (PSS‐78) with a handheld refractometer (precision 0.7%, accuracy 0.2%; Aqua Medic; http://www.aqua-medic.de) using *in situ* water samples. For Joal, monthly means in MODIS satellite‐derived (Aquarius, level 3, 0.5°) sea‐surface salinities were used.

### 2.2 Sample collection

Monthly sampling was conducted at the SSC and inside the Saloum River from February to October 2014, during *E. fimbriata*'s extended spawning season (Charles‐Dominique, 1982; Panfili *et al*., 2004). Three environmentally contrasted study sites were chosen: Joal (SSC, 14°9.1′ N; 16°51.7′ W), Djifer (Saloum River mouth, 13°57.8′ N; 16°44.8′ W) and Foundiougne (Saloum River middle reaches, 14°8.1′ N; 16°28.1′ W; Figure 1).

Fish were caught with gill nets (32–36 mm mesh size) by local fishermen and immediately stored on crushed ice after landing. Approximately 1000 fish per sampling site and month were examined randomly in order to find stage V females; *i.e*., mature individuals with ovaries containing fully hydrated oocytes (Blay Jr. & Eyeson, 1982). Sagittal otoliths were extracted, rinsed with ethanol (70%) and stored dry in Eppendorf caps. Females that spawned recently or lost part of their egg batch during handling were rejected.

### 2.3 Spawning energetics

Total wet mass (*M*_W_ ± 0.01 g) and total length (*L*_T,_ mm) were obtained from individual stage V females. Ovaries were dissected and oocytes were extracted out of one ovary lobe, rinsed with deionized water and counted under a stereomicroscope. Around 80 hydrated oocytes per fish (*c*. 70 for lipid analysis, *c*. 10 for protein analysis) were transferred to a pre‐weighed tin cap, stored in cryovials and deep‐frozen in liquid nitrogen. Dissected ovaries were transferred to a 4% borax buffered formaldehyde and freshwater liquid for fecundity analysis. Absolute batch fecundity (*B*_absfec_) was estimated gravimetrically using the hydrated oocyte method for indeterminate spawners (Hunter *et al*., 1985). The female's relative batch fecundity (*B*_relfec_) was calculated by dividing *B*_absfec_ with the ovary free body mass (*B*_absfec_ *M*_ovfree_^−1^; Alheit, 1988) (for details see Döring *et al*., 2017).

Oocytes in tin cups were freeze‐dried (24 h) and weighed again to ascertain their dry masss (*M*_OD_ ± 0.1 μg). Oocyte lipid content was determined by gas chromatography flame ionization detection (GC‐FID). Three random samples were processed *via* GC mass spectrometry to ensure that all lipid classes were detected by the GC‐FID. The total lipid content was assessed *via* summation of all individual lipid classes (for details see Döring & Ekau, 2017). For protein analyses, counted oocytes in tin cups were dried at 40°C for >24 h and weighed again for dry mass determination. Total organic carbon (C) and nitrogen (N) content was measured using a EuroVector EuroEA3000 elemental analyser (http://www.eurovector.it). From the total N in the sample, the protein content was calculated according to Kjeldahl (Bradstreet, 1954), using a nitrogen–protein conversion factor of 6.25. Oocyte lipid content was determined for 57 females, whereas oocyte protein content was measured for 67 females.

The oocyte gross energy (J) content was calculated on the basis of measured protein and lipid content, which were multiplied by corresponding energy values from literature: The amount of proteins per given oocyte (*M*_prot_, mg) was multiplied by a factor of 23.66 J mg^−1^ and subsequently added to the total amount of lipids per oocyte (*M*_lip_, mg) multiplied by 39.57 J mg^−1^ (Henken *et al*., 1986). Dividing the oocyte's energy content by the oocyte's dry mass allowed for calculation of the oocyte's calorific value (J mg^−1^).

The oocyte energy content of each individual *E. fimbriata* female was multiplied by its respective relative batch fecundity (*B*_absfec_) to obtain a standardized estimate of the total amount of energy invested in a single spawning batch per unit ovary‐free body mass (*E*_SB_, J g^−1^ *M*_OF_; Döring *et al*., 2018). The *E*_SB_ (J mg^−1^) was calculated for 52 females, where

$$E_{\mathrm{SB}}=\left(\left(23.66M_{\text{prot}}\right)+\left(39.57M_{\mathrm{lip}}\right)\right)B_{\text{relfec}}\;$$

### 2.4 Otolith elemental analyses

**Figure 2:**
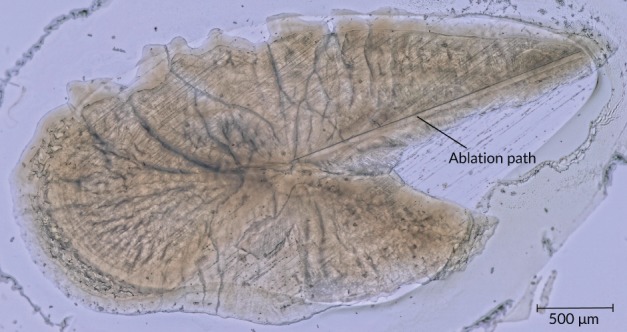
Ground otolith of a female *Ethmalosa fimbriata* specimen (total length, *L*_T_ = 23.1 cm) embedded in epoxy resin. The specimen was sampled at Foundiougne (Sine Saloum, Senegal) in May 2014. The ablation path can be seen along the edge of the otolith's rostrum

Dried sagittal otoliths were embedded in epoxy resin (Araldite 2020; http://www.go-araldite.com) on glass slides. They were ground from the proximal side down to the nucleus using an MPS2 surface‐grinding machine (G&N; http://www.grinders.de) and polished with a diamond‐grinding wheel (grain size 15 μm). Concentrations of eight elements (magnesium, manganese, copper, zinc (Zn), strontium (Sr), yttrium, barium (Ba) and lead) were determined along transects of up to 2300 μm length along the rostrum's anterior edge on the otolith's proximal side (Figure 2). Analyses were carried out by laser ablation inductively coupled plasma mass spectrometry (LA‐ICP‐MS) at the Department of Geosciences, University of Bremen, using a NewWave UP193 solid‐state laser coupled to a Thermo‐Finnigan Element2 ICP‐MS (http://www.thermofisher.com). The employed analytical procedure used a pulse rate of 10 Hz, an irradiance of *c*. 1 GW cm^−2^, a spot size of 75 μm and a traverse speed of 3 μm s^−1^ (Marohn *et al*., 2011). For external calibration the glass reference material NIST610 was analysed after every second ablation path, using reference values of Jochum *et al*. (2011). Data quality was validated by analysing a pressed pellet of NIES22 otolith (National Institute of Environmental Studies, Japan; http://www.nies.go.jp) with a calcium concentration of 38.8 wt. % (Yoshinaga *et al*., 2000), as well as through regular analyses of BCR‐2G and BHVO‐2G standard glasses (Jochum *et al*., 2005). Results showing standard measurement are given in Supporting Information Table S1. Precision was <2% and the accuracy was <13% for Zn. Recovery percentages were 100%, 101% and 114% for Ba, Sr and Zn, respectively. To account for the substitution of Calcium (Ca) by the divalent elements Ba, Sr and Zn all element concentrations are given as element:Ca ratios (Campana, 1999). Elemental analysis was conducted on the otoliths of 30 female fish and mean otolith Ba:Ca, Sr:Ca and Zn:Ca ratios were employed to discriminate between spawning sites. Differences in these three element‐to‐Ca ratios are commonly examined in studies on shad migration behaviour and stock delineation (Avigliano *et al*., 2017, Limburg, 1995, Magath *et al*., 2013, Rohtla *et al*., 2017, Secor & Rooker, 2000).

### 2.5 Statistical analyses

One‐way analysis of variance (ANOVA) and Tukey honest significant difference (HSD) *post hoc* tests were carried out to test for significant differences in oocyte energy content, oocyte calorific value and spawning batch energy content between sampling months and sampling sites. Since there were no site‐specific patterns in the whole‐life transects apparent (Supporting Information Figure S1), ANOVA was also used to test for spatial differences in mean elemental concentrations (Ba:Ca, Sr:Ca and Zn:Ca). Previously, normality (Shapiro‐Wilk test) and homogeneity of variance (Levene test) were tested. The oocyte energy content variable did not meet the homogeneity assumption and was square‐root transformed. The bivariate relationships between, oocyte lipid–protein content and *M*_OD_, as well as between element:Ca, temperature and salinity were tested for significance using linear‐regression analysis. To test for direct effects of environmental variables on the otolith element‐to‐Ca concentrations, the mean values of the last 30 μm of the traverse (otolith edge) were applied in linear‐regression analysis. Further, linear‐regression analysis revealed no significant effect of female length on any of the tested variables. Outliers were identified using the outlier boxplot function in JMP (SAS Institute Inc.; http://www.jmp.com). The significance level for all tests was set to 5%. All mean values are given ± SD.

Since no informed prior probabilities could be provided, a quadratic discriminant function analysis (QDFA) in combination with uniform priors was performed. This allowed for construction of a cross‐classification matrix to ascertain the capacity of the variables in question (oocyte calorific value, *E*_SB_, Ba:Ca, Sr:Ca and Zn:Ca) to identify the spawning site of the sampled females (White & Ruttenberg, 2007). Because of small and unequal sample sizes, Pillai's trace test was used to determine if the classification success rate was significantly different from random (Finch, 2005). Multicollinearity between mean element:Ca ratios was analysed using variance inflation factor (VIF < 2), a false outcome in the QDFA and the use of redundant variables in the study were therefore averted. The accuracy of the final predictive model was assessed using leave‐one‐out‐cross‐validation. The 22 individual females for which all parameters could be assessed were used as input for the discriminant analysis. All statistical analyses were carried out using JMP 10.0.1.

## 3 RESULTS

### 3.1 Female length distributions

**Figure 3:**
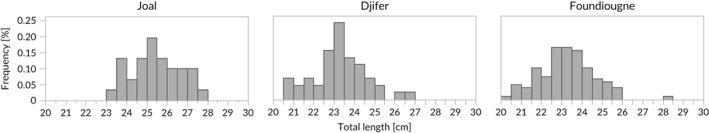
Total length–frequency distribution of *Ethmalosa fimbriata* females with hydrated oocytes. Sampling took place at Joal (Senegalese southern coast), Djifer (Saloum River mouth) and Foundiougne (Saloum River middle reaches) from February to October 2014

*Ethmalosa fimbriata* females with ovaries containing hydrated oocytes were sampled in the size ranges 23.1–27.7, 20.8–26.9 and 20.1–28.3 cm at Joal (SSC), Djifer (Saloum River mouth) and Foundiougne (Saloum River middle reaches), respectively. Within the observed distributions, the modes of the highest frequency were *c*. 25.5 cm at Joal and 23.5 cm at Djifer and Foundiougne (Figure 3).

### 3.2 Spawning energetics

**Figure 4:**
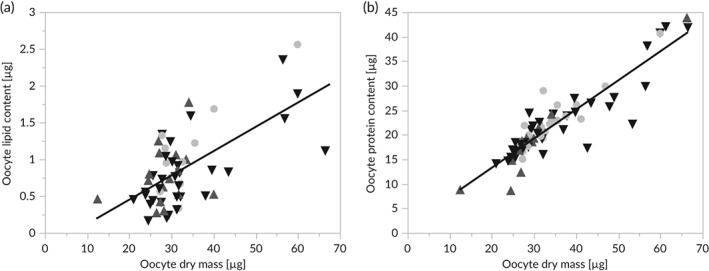
Relationships between *Ethmalosa fimbriata* oocyte dry mass and (a) lipid content, and (b) protein content from fish in southern Senegalese coastal waters. Specimens were sampled at 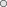 Joal (Senegalese southern coast), 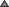 Djifer (Saloum River mouth) and 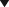 Foundiougne (Saloum River middle reaches) from February to October 2014

**Figure 5:**
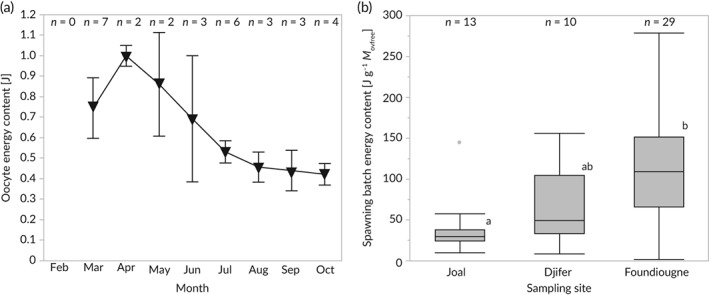
(a) Monthly mean (±SD; *n*, sample size) oocyte energy content and (b) boxplots (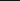, median; ⊺, 5th and 95th percentiles; , outliers) of spawning batch energy content of female *Ethmalosa fimbriata* sampled at Joal (Senegalese southern coast), Djifer (Saloum River mouth) and Foundiougne (Saloum River middle reaches) from February to October 2014. Different lowercase letters indicate significant differences (*P* < 0.05). *M*_ovfree_, Ovary‐free body mass

Mean absolute oocyte lipid content was determined to be 0.9 ± 0.5 μg, corresponding to 2.7 ± 1.3% of oocyte dry mass. A mean protein content of 22.2 ± 7.5 μg or 64.6 ± 5.0% of the oocyte dry mass was recorded. Linear relationships between oocyte absolute lipid content and *M*_OD_ (*F*_1,56_ = 38.6, *P* < 0.0001; *r* ^2^ = 0.41; Figure 4(a)), as well as between oocyte absolute protein content and *M*_OD_ were identified (*F*_1,66_ = 301.1, *P* < 0.001, *r* ^2^ = 0.82; Figure 4(b)). The energy content of single oocytes spawned at Foundiougne (Saloum River middle reaches) was significantly different among sampling months (ANOVA *F*_7,29_ = 6.5, *P* < 0.001, Figure 5(a)). Oocyte energy content was significantly higher in March–May than in October 2014 (Tukey HSD *P* < 0.05). No monthly differences in oocyte energy content were observed at Joal (ANOVA *F*_2,12_ = 1.6, *P* > 0.05) and at Djifer (ANOVA *F*_3,9_ = 1.1, *P* > 0.05). Furthermore, no significant spatial or temporal differences in oocyte calorific values were detected. The mean value across all sampling sites was determined to be 15.2 ± 1.4 J mg^−1^. No significant monthly differences in energy invested into one spawning batch (*E*_SB_) were detected. Still, female *E. fimbriata* sampled at Foundiougne invested significantly more energy into spawning when compared with their counterparts sampled at Joal (ANOVA *F*_2,51_ = 8.9, *P* < 0.001; Tukey HSD *P* < 0.05; Figure 5(b)).

**Figure 6:**
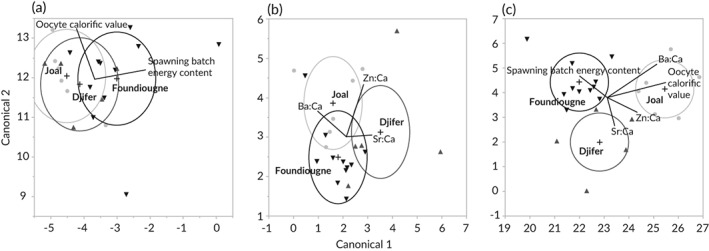
Quadratic discriminant function analysis of the (a) spawning energetics, (b) otolith element:Ca ratios and (c) both techniques combined in female *Ethmalosa fimbriata* sampled at 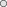 Joal (Senegalese southern coast), 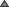 Djifer (Saloum River mouth) and 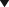 Foundiougne (Saloum River middle reaches) from February to October 2014. Ellipsoids encompass *c*. 50% of a sampling station's data points

The QDFA plot employing the seasonally unaltered reproductive traits oocyte calorific value and *E*_SB_ (Figure 6(a)) showed little separation between sampling sites. The QDFA cross‐classification matrix (Table 1) revealed a high to moderate percentage of correctly classified individuals for Joal (100%), Djifer (40.0%) and Foundiougne (45.6%), although not significantly different from random (Pillai's trace test: *P* > 0.05), providing no discriminatory power.

**Table 1:** Cross‐classification matrix of the quadratic discriminant analysis employing female *Ethmalosa fimbriata* spawning energetics (oocyte calorific value and spawning‐batch energy content), otolith microchemistry (Ba:Ca, Sr:Ca and Zn:Ca), as well as both techniques combined

	Joal	Djifer	Foundiougne	Sample size (*n*)	Misclassified (%)	–2logLikelihood	*P*
Spawning energetics					40.9	32.8	>0.05
Joal, Senegalese southern coast	**100**	0	0	6			
Djifer, Saloum River mouth	40.0	**40.0**	20.0	5			
Foundiougne, Saloum River middle reaches	9.1	45.6	**45.6**	11			
Otolith microchemistry					22.7	18.2	<0.05
Joal	**83.3**	0	16.7	6			
Djifer	0	**60.0**	40.0	5			
Foundiougne	9.1	9.1	**81.8**	11			
Spawning energetics + Otolith microchemistry					9.1	5.0	<0.001
Joal	**100**	0	0	6			
Djifer	0	**100**	0	5			
Foundiougne	0	18.2	**81.8**	11			

Correctly classified fish per sampling site are in bold.

### 3.3 Otolith microchemistry

Otolith mean Ba:Ca values throughout the entire lifetime of the female fish were significantly higher in individuals sampled at Joal than at Djifer and Foundiougne (ANOVA, *F*_2,27_ = 7.5, *P* < 0.01). Also, otolith mean Zn:Ca values were significantly higher at Joal than at Foundiougne, with otoliths of females sampled at Djifer exhibiting intermediate values (ANOVA, *F*_2,27_ = 5.2, *P* > 0.05). No significant differences in otolith mean Sr:Ca content could be observed among areas (ANOVA, *F*_2,29_ = 1.3, *P* > 0.05).

**Figure 7:**
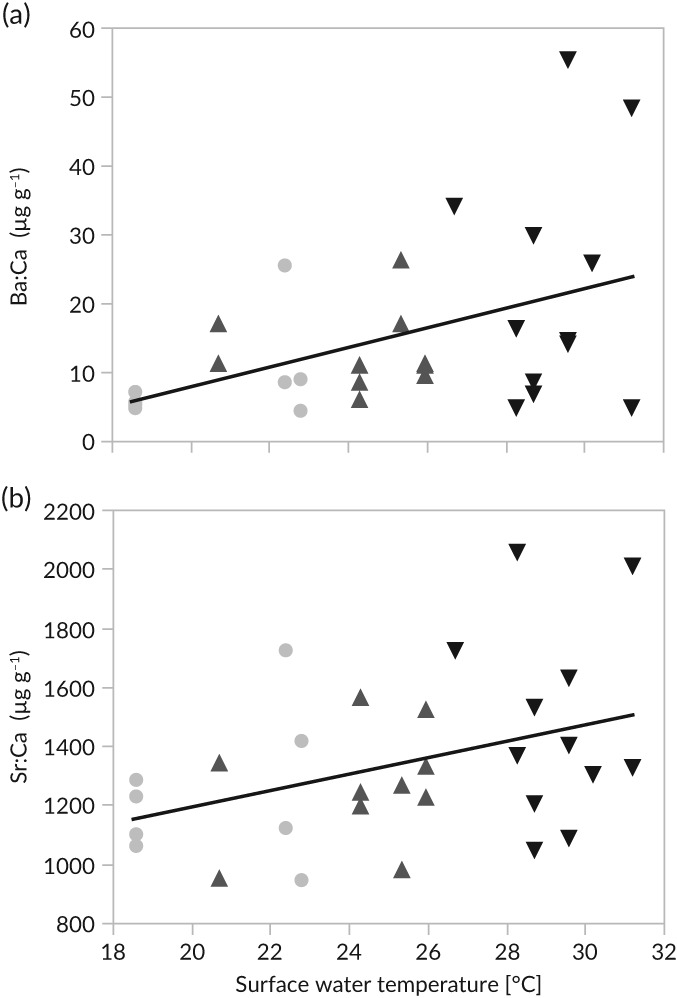
Mean (a) Ba:Ca and (b) Sr:Ca ratios of female *Ethmalosa fimbriata* otoliths in relationship with surface water temperature. Individuals were sampled at 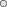 Joal (Senegalese southern coast), 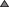 Djifer (Saloum River mouth) and 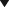 Foundiougne (Saloum River middle reaches) from February to October 2014

Mean Ba:Ca (linear regression, *F*_1,29_ = 6.9, *P* > 0.05; *r* ^2^ = 0.20; Figure 7(a)) and Sr:Ca (linear regression, *F*_1,29_ = 5.1, *P* < 0.05; *r* ^2^ = 0.15; Figure 7(b)) of the 30 μm closest to the otolith's edge were positively correlated with surface‐water temperature. No linear relationships between environmental variables and Zn:Ca were observed.

All three elements are commonly used to discriminate between fish populations and were consequently employed for stock spawning component discrimination using QDFA. The QDFA plot (Figure 6(b)) showed little separation between sampling sites. The cross‐classification matrix (Table 1) revealed a moderate percentage of correctly classified individuals (83.3%, 60.0% and 81.8% for Joal, Djifer and Foundiougne, respectively), with 22.7% individuals misclassified, while significantly different from random (Pillai's trace test *P* < 0.05).

### 3.4 Identification of stock spawning components

The QDFA plot combining both classification techniques showed a clear separation between sampling sites (Figure 6(c)). The cross‐classification matrix integrating spawning energetics and otolith microchemistry yielded 90.9% correctly classified individuals, while significantly different from random (Pillai's trace test, *P* < 0.001; Table 1). Cross‐validation revealed a mean of 7.1 ± 3.2% misclassified individuals with a mean ‐2logLikelihood of 6.9 ± 4.3.

## 4 DISCUSSION

Our results show that the combined use of spawning energetics and otolith microchemistry in the discriminant function analysis yields high classification percentages and thus illustrate distinct spawning stock components of Senegalese *E. fimbriata*. A variety of reproductive life‐history traits have been used to distinguish between stock spawning components, including timing, duration and location of spawning; age, length and mass at maturity; egg mass, size, viability and fecundity relationships; proportion of recruit and repeat spawners; and maternal effects as well as reproductive potential (Jakobsen *et al*., 2009). Also, assessments of the energy invested in reproduction are of ever‐growing concern since this information is advantageous for stock‐component productivity appraisal and for bio‐energetic model construction (Pecquerie *et al*., 2009). In recent years, the focus shifted increasingly towards elemental analysis of fish otoliths in order to discriminate among stock components (Kerr & Campana, 2014).

*Ethmalosa fimbriata*'s Senegalese and Gambian stocks are thought to be one population with an age‐specific habitat use (Charles‐Dominique & Albaret, 2003). Juveniles are thought to use estuaries as their main nursery ground (Gning *et al*., 2007) and then emigrate to the sea as adults (Charles‐Dominique & Albaret, 2003). This hypothesis could not be supported by the length frequency distributions obtained throughout the sampling sites at the Senegalese coast and within the Sine Saloum Estuary. Spatial variations in spawner length distributions were negligible and may rather originate from differences in size‐selectivity of gill nets used by local fishermen (Faye *et al*., 2018).

Assessment of oocyte lipid and protein content allows for proper estimation of energy supply for embryonic development through the egg yolk (Kamler, 2005). Across all sampling sites (Joal: SSC, Djifer: Saloum River mouth, Foundiougne: Saloum River middle reaches), a linear correlation of oocyte lipid, as well as protein content with oocyte dry mass, was observed. The nutritional value of a given oocyte is, therefore, increasing with its mass. In clupeids heavier, nutritionally‐rich oocytes are usually produced at the beginning of the spawning season, when water temperatures are comparatively low and developmental times in early life stages are long due to metabolic constraints (Bradford & Stephenson, 1992; Riveiro *et al*., 2004). *Ethmalosa fimbriata* exhibits spawning in the hypersaline middle reaches of the Saloum River all year around (Panfili *et al*., 2004) and here the dry masses of their hydrated oocytes were shown to be significantly higher during the dry season than during the wet season (Döring & Ekau, 2017). In the current study, mean energy content of single hydrated oocytes of females sampled at Foundiougne were found to be significantly higher during the cold and dry season (March–May) than at the end of the wet season (October). This may be a valuable adaptation to longer ontogenetic times due to lower water temperatures at the beginning of the year and to elevated metabolic demands due to overall high salinities in the estuary's middle reaches during the dry season (Alderdice & Forrester, 1968; Alderdice & Velsen, 1971; Jatteau *et al*., 2017).

While batch fecundities in females sampled at Foundiougne were significantly increasing from February to October (Döring *et al*., 2017), the energy invested in a single spawning batch (*E*_SB_) did not vary temporally. Thus, *E. fimbriata* spawned more eggs but of lower dry mass/energy content at Foundiougne during the wet season and *vice versa* during the dry season (Döring & Ekau, 2017). Temporal trade‐offs between fecundity and oocyte energy content seem to be a viable spawning tactic in clupeid fishes to ensure early life‐stage survival (Castro *et al*., 2009; Döring *et al*., 2018). Still, significant spatial differences in *E*_*SB*_ were encountered between individuals reproducing at the SSC and their counterparts spawning inside the Saloum River.

QDFA of spatially resolved spawning energetics yielded no significant result. The fact that Saloum River individuals invested three times more energy into reproduction, however, indicates higher productivity of the estuarine population. Apart from distinct spawning periods, former studies determined diverging values for the length at first maturity and differences in batch fecundity between the estuarine and the coastal population (Charles‐Dominique & Albaret, 2003; Döring *et al*., 2017; Panfili *et al*., 2004; Scheffers *et al*., 1972). Differences in these spawning traits are probably a result of variations in fishing pressure and adaptations to diverging hydrographic conditions (Jakobsen *et al*., 2009). In any case, varying individual reproductive investment ultimately alters a stock's productivity and consequently has been the basis of past stock spawning‐component discrimination in clupeids (Bradford & Stephenson, 1992). Our results, therefore, suggest that there are two productively distinct spawning components, one at the coast and one within the Sine Saloum estuary.

The sampling sites at the SSC and inside the Saloum River obviously have specific hydrographic dynamics. Annually reoccurring seasonal upwelling at the SSC affects water temperatures as well as chlorophyll levels/phytoplankton abundances (Ndoye *et al*., 2014, 2017; Tiedemann & Brehmer, 2017). At the Saloum River's mouth, tides govern the exchange of water bodies and inside the Saloum River's middle reaches high evaporation rates and little precipitation alter the hydrographic conditions throughout the year (Pagès & Citeau, 1990). The water characteristics potentially print distinct signatures in the otoliths if females spent a significant amount of time (*e.g*., for spawning) within one of these habitats (Avigliano *et al*., 2017). In the current study significant positive correlations of otolith Ba:Ca and Sr:Ca levels with surface water temperatures at the time of capture could be observed. Also, significant spatial differences in Ba:Ca and Zn:Ca give strong evidence of well‐differentiated female habitat usage in the examined populations. The cross‐classification matrix yielded percentages of correctly classified individuals that are marginally smaller than in a study conducted by Avigliano *et al*. (2017) on estuarine streaked prochilod *Prochilodus lineatus* (Valenciennes, 1837) using the same elements.

Some marine fish stocks comprise several spawning components that may show over‐lapping distributions (Jakobsen *et al*., 2009). The QDFA integrating spawning energetics and otolith microchemistry yielded high classification success. Its application to *E. fimbriata* in southern Senegalese waters, therefore, resulted in the detection of spatially distinct spawning components. These findings are consistent with observations made in past studies (Charles‐Dominique, 1982; Charles‐Dominique & Albaret, 2003; Durand *et al*., 2013; Panfili *et al*., 2006) and the small geographical scale (15–45 km) on which population fractionation could be detected was comparable with the one reported for American shad *Alosa sapidissima* (Wilson 1811) (Melvin *et al*., 1992). The clear distinction between spawning components at the coast, the Saloum River's mouth and inside the river's middle reaches is most likely due to the estuary's distinct hydrological characteristics, which are markedly different from neritic conditions. Heterogeneous hydrographic features lead to incorporation of varying element:Ca ratios into the female spawners’ otoliths and further require energetical adaptations to ensure recruitment success (Avigliano *et al*., 2017; Döring *et al*., 2018). As the low sampling sizes in the QDFA models suggest, assessing all investigated variables for each individual fish is challenging. Standardising both methodologies to analyse spawning energetics and otolith elemental fingerprint of clupeid fishes would improve the effort and simplify the execution. Given the high classification success of the final model, the approach presented here of combining spawning energetics and otolith elemental fingerprints is a valuable method for the identification of spawning components in clupeid fish stocks.

Investigation of reproductive biology provides immediate insights into the processes responsible for the formation and maintenance of fish‐stock structure. The basis of the biological definition of a stock is that they are reproductively isolated units, with individuals of each potential stock exhibiting homogeneous traits. Conversely, information on the stock structure is essential for understanding reproductive patterns. An individual female's ability to invest energy into reproduction is closely coupled with the stock's reproductive potential and thus with its productivity. Significant spatial differences in reproductive investment and otolith element:Ca profiles in Senegalese *E. fimbriata* could be identified. It is evident that there are several spawning components in southern Senegalese waters that are characterised by delimited home ranges and distinctive productivity. Less productive components; *i.e*., *E. fimbriata*'s neritic sub‐population, are more likely to become overexploited and a decline in catch rates could ultimately compromise the fisheries’ activity and affect the depending socio‐economic sector. In order to preserve reproductive potential over the stock's full geographical range, specialized management approaches such as spatially variable time closures are recommended (Sadovy *et al*., 2003; Sadovy & Cheung, 2003).

## Supporting information


**Figure S1** (a) Ba:Ca, (b) Sr.:Ca, (c) Zn:Ca ratios along sagittal otolith transects from the edge to the nucleus in *Ethmalosa fimbriata* females sampled at Joal (Senegalese south coast), Djifer at the (Saloum River mouth) and Foundiougne (Saloum River middle reaches) in April 2014. (μg g^−1^).

**Table S1** Results for the elemental analyses (μg g^−1^) of the three different standards used for validation. NIES‐22 was only measured once, measured mean values for BCR‐2G and BHVO‐2G are given including standard deviations.
