# Supplementary material for: Spawning energetics and otolith microchemistry provide insights into the stock structure of bonga shad Ethmalosa fimbriata
Source: J Fish Biol. 2019 Jan 15;94(2):241–50. doi: 10.1111/jfb.13881 (PMC6849771; doi:10.1111/jfb.13881)
Supplement: Supplementary file 2 — Table S1 Results for the elemental analyses (μg g−1) of the three different standards used for validation. NIES‐22 was only measured once, measured mean values for BCR‐2G and BHVO‐2G are given including standard deviations. [file JFB-94-241-s002.docx]

**Appendix**

**Appendix Table 1** Results for the elemental analyses (µg g^-1^) of the three different standards used for validation. NIES-22 was only measured once, measured mean values for BCR-2G and BHVO-2G are given including standard deviations.

| Element | NIES-22 | | BCR-2G | | BHVO-2G | |
| --- | --- | --- | --- | --- | --- | --- |
|  | Reference value | Measured value | Reference value | Measured value | Reference value | Measured value |
| Mn |  | 0.121 | *1550* | 1500±65 | *1317* | 1364±62 |
| Mg | *21.0* | 17.6 | *21467* | 18963±346 | *42996* | 39771±460 |
| Ba | *2.89* | 2.53 | *683* | 667±16 | *131* | 133±3 |
| Cu | *0.740* | 0.721 | *21.0* | 16.9±0.3 | *127* | 124±3 |
| Pb | *0.0230* | 0.0308 | *11.0* | 10.2±0.4 | *1.70* | 1.71±0.1 |
| Sr | *2360* | 2308 | *342* | 339±5 | *396* | 405±8 |
| Y |  | 0.00898 | *35.0* | 33.5±0.6 | *26.0* | 24.6±0.4 |
| Zn | *0.470* | 0.560 | *125* | 146±3 | *102* | 113±2 |
|  |  |  |  |  |  |  |
| Nr. of measurements | 1 | | 6 | | 7 | |
